# Structure-guided fragment-based drug discovery at the synchrotron: screening binding sites and correlations with hotspot mapping

**DOI:** 10.1098/rsta.2018.0422

**Published:** 2019-04-29

**Authors:** Sherine E. Thomas, Patrick Collins, Rory Hennell James, Vitor Mendes, Sitthivut Charoensutthivarakul, Chris Radoux, Chris Abell, Anthony G. Coyne, R. Andres Floto, Frank von Delft, Tom L. Blundell

**Affiliations:** 1 Department of Biochemistry, University of Cambridge, Tennis Court Road, Cambridge CB2 1GA, UK; 2 Diamond Light Source (DLS), Harwell Science and Innovation Campus, Didcot OX11 0DE, UK; 3 Sir William Dunn School of Pathology, University of Oxford, South Parks Road, Oxford OX1 3RE, UK; 4 Department of Chemistry, University of Cambridge, Lensfield Road, Cambridge CB2 1EW, UK; 5 The European Bioinformatics Institute (EMBL-EBI), Wellcome Genome Campus, Cambridge CB10 1SD, UK; 6 Cambridge Centre for Lung Infection, Royal Papworth Hospital, Cambridge CB23 3RE, UK; 7 Molecular Immunity Unit, Department of Medicine, University of Cambridge, MRC-Laboratory of Molecular Biology, Cambridge CB2 0QH, UK; 8 Structural Genomics Consortium, Nuffield Department of Medicine, University of Oxford, Oxford OX3 7DQ, UK; 9 Department of Biochemistry, University of Johannesburg, Auckland Park 2006, South Africa

**Keywords:** synchrotron, structure-guided, fragment-based drug discovery, SAICAR synthetase (PurC), *Mycobacterium abscessus*

## Abstract

Structure-guided drug discovery emerged in the 1970s and 1980s, stimulated by the three-dimensional structures of protein targets that became available, mainly through X-ray crystal structure analysis, assisted by the development of synchrotron radiation sources. Structures of known drugs or inhibitors were used to guide the development of leads. The growth of high-throughput screening during the late 1980s and the early 1990s in the pharmaceutical industry of chemical libraries of hundreds of thousands of compounds of molecular weight of approximately 500 Da was impressive but still explored only a tiny fraction of the chemical space of the predicted 10^40^ drug-like compounds. The use of fragments with molecular weights less than 300 Da in drug discovery not only decreased the chemical space needing exploration but also increased promiscuity in binding targets. Here we discuss advances in X-ray fragment screening and the challenge of identifying sites where fragments not only bind but can be chemically elaborated while retaining their positions and binding modes. We first describe the analysis of fragment binding using conventional X-ray difference Fourier techniques, with *Mycobacterium abscessus* SAICAR synthetase (PurC) as an example. We observe that all fragments occupy positions predicted by computational hotspot mapping. We compare this with fragment screening at Diamond Synchrotron Light Source XChem facility using PanDDA software, which identifies many more fragment hits, only some of which bind to the predicted hotspots. Many low occupancy sites identified may not support elaboration to give adequate ligand affinity, although they will likely be useful in drug discovery as ‘warm spots’ for guiding elaboration of fragments bound at hotspots. We discuss implications of these observations for fragment screening at the synchrotron sources.

This article is part of the theme issue ‘Fifty years of synchrotron science: achievements and opportunities’.

## 1. Background

Structure-guided drug discovery has its origins in both academia and the pharmaceutical industry in the 1970s (for reviews, see [1,2]). The need to modify and elaborate natural compounds and other molecules found to inhibit target proteins began to stimulate interest in the crystal structures of proteins that were becoming available [3]. The structural information of aspartic proteinases such as renin as a target for anti-hypertensives [4,5] and HIV protease in the 1980s for AIDS [6,7] demonstrated the value of detailed knowledge of protein–ligand interactions in the design of new compounds. In parallel in the late 1980s and 1990s, the development of high-throughput screening (HTS) led to the construction of chemical libraries of millions of compounds. However, the huge size and diversity of ‘chemical space’, estimated to be 10^40^ molecules for drug-like compounds of molecular weights of approximately 500 Da, began to be a focus, as the pharma industry realized that existing large chemical libraries explored only a very small part of chemical space. An alternative approach to the challenge was found in decreasing the size of the molecules from the molecular weight of approximately 500 Da to less than 300 Da, which not only decreased the size of chemical space needing exploration but at the same time increased their promiscuity in binding targets. This laid down the basic principles of fragment-based drug discovery (FBDD) [see [2], for review].

Early fragment screening approaches included those at Abbott using ligand-based nuclear magnetic resonance [8] and at Astex using X-ray analysis [9], developed initially by exploiting high-throughput analysis of cocktails of six to ten fragments soaked into apo-protein crystals [10]. Knowledge of the structure of the complex of the fragment with the target protein allowed the initial use of small, often non-chiral compounds, which were optimized using structure-guided approaches to make specific interactions and to introduce chirality into the molecules. The fragment hits were capable of achieving high binding efficiency per atom and often better physico-chemical properties in comparison to those from HTS, which exploits much larger libraries of approximately 10^5^ or even 10^6^ compounds.

With encouragement and funding from the Bill and Melinda Gates Foundation in 2006, the structure-guided FBDD developed in Cambridge was spun back from Astex into the University with an initial focus on targeting *Mycobacterium tuberculosis* resulting in some success in producing lead and candidate molecules [11]. Structure-guided FBDD is particularly well suited to academia in requiring inexpensive fragment libraries and depending on molecular biology, preparative biochemistry, structural, computational and biophysical methods available in academic structural-biology laboratories. This encouraged the extension of its use in targeting other mycobacterial targets including *Mycobacterium abscessus*, an increasing problem for cystic fibrosis patients, and *Mycobacterium leprae* where leprosy remains a major challenge in many parts of the world, with 211 973 new cases reported globally in 2015 [12].

During the past four decades, synchrotron radiation facilities have played an increasingly central role in structure-guided drug discovery. The pharmaceutical industry was initially sometimes hesitant to exploit the facilities, because they concerned crystals involving compounds with large intellectual property (IP) value to be sent outside the company. In academia, this was less of a challenge, with the focus often being on early discovery rather than securing IP and in the study of neglected diseases, where the financial returns are unlikely to be great given their prevalence in developing countries or small patient populations. However, the pharmaceutical industry has become a major driver for increased automation at synchrotrons worldwide, often using beamlines built by individual companies. Along with continuous improvements in beam intensity, detector technology, robotic sample handling and data analysis software, the speed and accuracy of the diffraction experiments have been systematically transformed [13]. These developments have made it possible to make fragment screening by X-ray structure routinely and widely accessible.

A major advance has been the XChem facility at the Diamond synchrotron [14] which has implemented further streamlining of crystal preparation [15]. This development has been combined with the new Pan-Dataset Density Analysis (PanDDA) tool [16] that increases sensitivity, revealing fragments in even partially occupied binding sites by contrasting multiple unbound and ligand-bound-protein X-ray datasets to extract signals for bound fragments.

**Figure 1.**
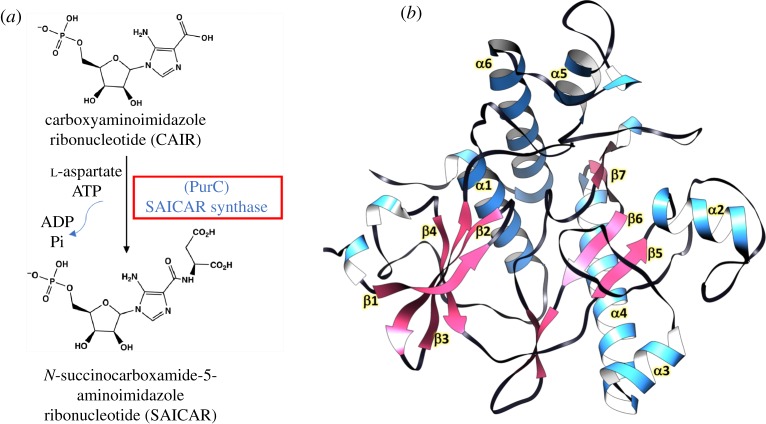
(*a*) Schematic depiction of the enzyme reaction catalysed by PurC (SAICAR synthetase) in the bacterial purine biosynthesis pathway. (*b*) Crystal structure of apo form of *M. abscessus* PurC refined at 1.5 Å resolution, coloured by secondary structure.

Although there has been intense use of XChem [14] and PanDDA software [16,17] at Diamond and an awareness that many more fragment binding sites tend to be identified, there has been little work on specific targets in comparing the new approach with the earlier one using standard difference Fourier X-ray analysis, usually assuming full occupancy of ligands on the same target protein. Here, we discuss the use of an ongoing structure-guided FBDD programme to compare the two approaches. The target selected, PurC, or phosphoribosylaminoimidazole-succinocarboxamide (SAICAR synthetase) from *M. abscessus*, is involved in the biosynthesis of purine nucleotides [18]. The enzyme catalyses the eighth step of the *de novo* purine biosynthesis pathway in bacteria and fungi, mediating the ligation of l-aspartate with 5-amino-1-(5-phospho-d-ribosyl) imidazole-4-carboxylate (CAIR) in the presence of adenosine 5′-triphosphate (ATP) and Mg^2+^ to form SAICAR, as shown in figure 1*a*. The importance of *de novo* purine biosynthesis in maintaining the viability of cells and differences in the structural architecture of bacterial and human PurC orthologues makes it an ideal target for antimicrobial agents [19–21], as further illustrated in the electronic supplementary material, figure S1.

In this study, we focus on the fragment binding modes of MabPurC defined by X-ray analysis at the synchrotron using the standard difference Fourier approach, following a preliminary screening of a fragment library using biophysical techniques such as differential scanning fluorimetry (DSF) and isothermal titration calorimetry (ITC). We then describe recent experiments on PurC at the Diamond light source at the Rutherford Laboratory using the high-throughput and roboticized X-ray screening method, XChem, developed by von Delft and colleagues [14]. This, together with PanDDA software [16,17], exploits the multiple apo-protein crystal structures from the synchrotron X-ray screening that have no fragments bound, resulting in a more accurate description of both the apo-enzyme structure and the fragment binding and occupancy in the complex.

We also compare the experimental fragment binding sites derived using the two X-ray approaches with those predicted by the use of Fragment Hotspot Maps [22], where hot spots are defined computationally by their ability to bind a small molecule fragment. The software exploits experience of fragment binding experiments over many years indicating that binding sites tend to have a polar donor and/or acceptor binding capability as well as non-polar regions in close proximity. It is assumed that this limits not only the translational but also the rotational entropy of water molecules at the sites in the apostructures, so making the release of the ‘unhappy’ waters more entropically favourable and their replacement by a fragment more favoured. The entropic gain in the release also tends to be further increased by the release of fragments from deeper pockets, where rotational freedom is further limited. These features were built into Fragment Hotspot Maps, developed by Radoux *et al.* [22].

Here, the focus is to compare the new developments in FBDD at XChem with those used over the past two decades, mainly aided by synchrotron radiation, using MabPurC as an example of a drug discovery target. We briefly describe the information about PurC activity and structure, which is necessary for understanding the fragment binding using the two experimental approaches. We do not discuss the next stages of fragment-linking or chemical elaboration, which are being pursued in parallel for PurC as a target for combatting infections by *M. abscessus* in cystic fibrosis.

## 2. Results

### (a) Overall structure of *M. abscessus* PurC and substrate binding

**Figure 2.**
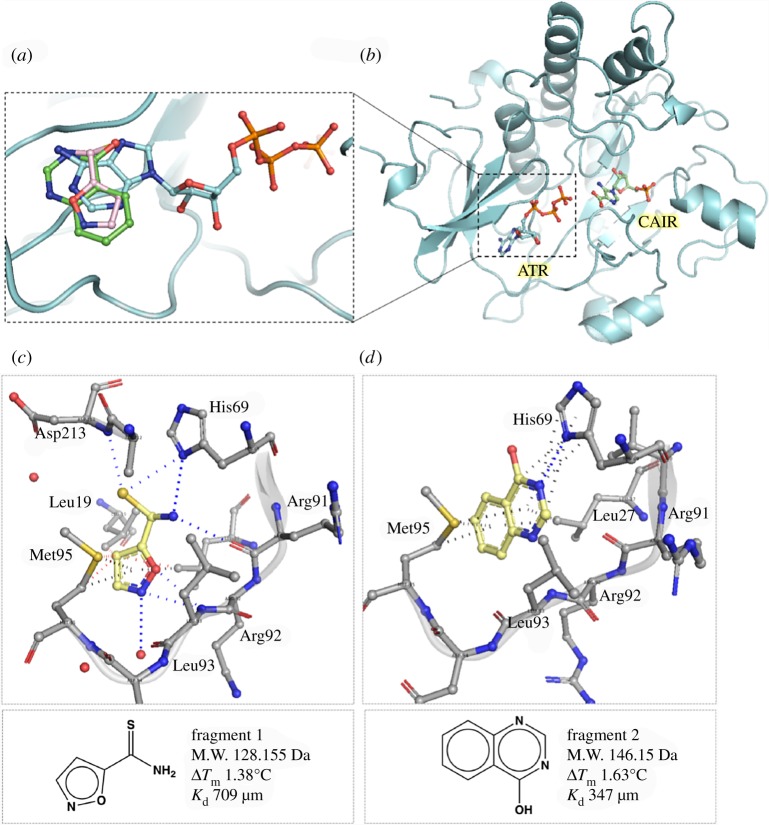
*M. abscessus* PurC in complex with natural ligands and fragment hits. (*a*) Crystal structure of Mab PurC in complex with ATP (blue) showing the position of fragments 1 (pink) and 2 (green) with respect to ATP adenine ring; (*b*) crystal structure of Mab PurC in complex with ATP (blue) and the inferred position of substrate CAIR (green) in MabPurC derived by superposition with *E. coli* PurC (PDB code 2GQS); (*c*) fragment 1 and (*d*) fragment 2, showing interaction of fragment hits (yellow stick) with residues at the ATP site (grey stick). Hydrogen bonding interactions are depicted in blue, π-interactions in black and hydrophobic contacts in red dotted lines, respectively. The corresponding two-dimensional structures of fragments and biophysical data are shown below.

The apo structure of MabPurC was solved and refined at 1.5 Å resolution. The crystals are similar to those of a previously determined structure of MabPurC with a monomer in the asymmetric unit (figure 1*b*) (PDB 3R9R, Seattle Structural Genomics Consortium for Infectious Diseases). *M. abscessus* PurC (MabPurC), like other PurC orthologues, is a globular protein consisting of two lobes spanning a long catalytic cleft. To investigate further the substrate interactions in the PurC catalytic cleft, the crystal structure of *M. abscessus* PurC in complex with ATP was solved at 1.2 Å resolution, following co-crystallization of the protein with ATP. The resulting structure shows that ATP occupies one end of the active site cleft with the phosphate groups extending towards a positively charged pocket towards the middle of the cleft. Superposition of the MabPurC: ATP structure with that of *E. coli* PurC: CAIR-bound form (PDB code 2GQS) shows that the catalytic cleft contains sites for ATP at one end and CAIR at the other (figure 2*b*). It has been proposed previously that l-aspartate may occupy the space between ATP and CAIR [23,24].

### (b) Initial fragment screening and characterization of hits

Our initial fragment screening approach used methods developed in Cambridge in Astex and in the University (for a review, see [11]). A library of 960 small molecule fragments was screened by DSF, resulting in 43 hits, which were then investigated by X-ray crystallography using the standard difference Fourier approach. MabPurC apo crystals were soaked with each fragment in individual experiments. The eight fragment hits identified from the resulting crystal structures were all found to occupy the ATP pocket of MabPurC, recapitulating key binding interactions of the adenine ring (figure 2; electronic supplementary material, table S1) in the structure of MabPurC.

As illustrated in figures 2*c*,*d*, the binding modes of fragments **1** and **2** are similar to those of the ATP adenine ring in this region. These include H-bond interaction to the His69 side chain and to the backbone amide nitrogens of Leu93 and Asp213 and backbone carbonyl of Arg91, and many water-mediated hydrogen bonds in the active site. π-interactions of the fragments are mainly mediated by the side chain of Met95 at the edge of the active site cleft. Many fragments also engage in stacking and hydrophobic interactions with the side chains of Leu27 at the top of the cleft. However, marked differences were observed in the orientation of the planar ring of the fragments at the adenine pocket depending on the ring variant and chemical substitutions. The binding interactions of all the above fragment hits with MabPurC were further characterized by ITC and the calculated *K*_d_ values ranged from 178 to 971 µM (electronic supplementary material, table S1).

### (c) Computational hotspot mapping of *M. abscessus* PurC

The MabPurC structure was further analysed using Fragment Hotspot Maps [22], with the objective of investigating the binding propensities of ligands. Work with *M. abscessus*, *M. leprae* and *M. tuberculosis* (Thomas SE, Mendes V, Vedithi SC and Blundell TL, unpublished) indicates that the software is able to reproduce natural ligand sites when using contour levels of 17 and above. Contour level 14 additionally reveals ‘warm spots’ where a fragment may not bind but where further interactions can stabilize a ligand as it is grown from a fragment bound to an adjacent hotspot.

**Figure 3.**
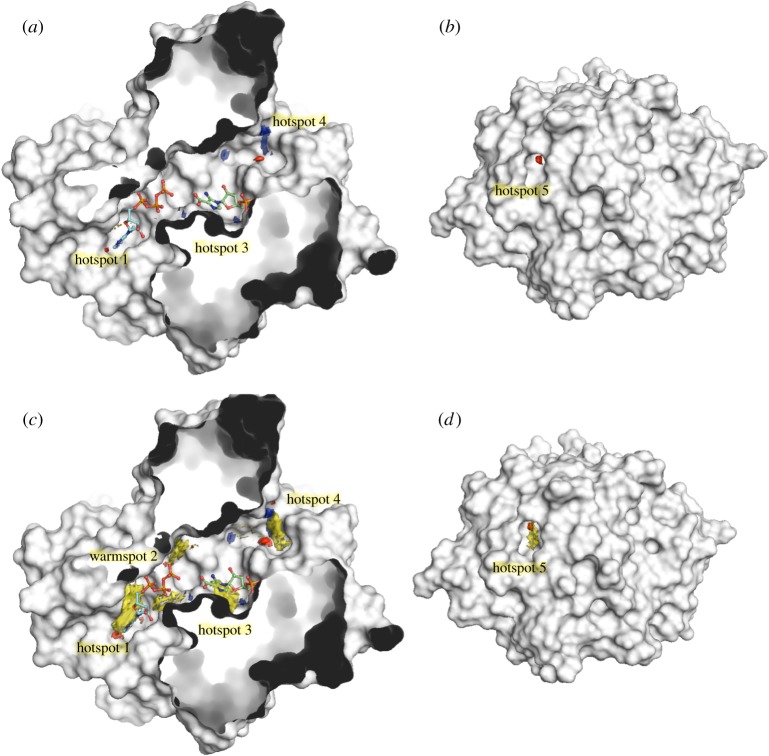
Hotspot mapping of PurC. Crystal structure of MabPurC protein (white), shown in surface representation, with hotspot maps showing hydrogen bond donor (blue), acceptor (red) and hydrophobic (yellow) regions. ATP (blue stick) and CAIR (green stick) are also shown. (*a*) Three hotspot regions are observed at the active site cleft at the front, when the maps are contoured at 17. (*b*) A fifth hotspot consisting of an acceptor region is seen at the rear of the protein. (*c*) A warm spot (warm spot 2) can also be seen in addition to the three hotspots, when the maps are contoured at 14 and the hydrophobic patches (yellow) at all the hotspot regions become more prominent at this contour. (*d*) The fifth hotspot at the rear of the protein when observed at contour 14. The hotspot maps were generated as described in [22].

Superposition of the resulting hotspot map, contoured at 17, with the previously defined ATP- and CAIR-bound structures show three hotspot regions within the active site cleft (figure 3*a*) and an additional small hotspot pocket on the distal side (figure 3*b*). The first hotspot is in the ATP adenylyl-binding region, with residues ^91^RRLDM^95^ and His 69 providing three H-bond acceptor and one donor interactions. Most fragments that bind in this pocket also satisfy the hydrogen bond donor (blue), acceptor (red) and hydrophobic (yellow) interactions of hotspot 1.

When the same map is observed at contour 14, an additional ‘warm spot’ (warm spot 2) region can be seen adjacent to the flexible loop and β-hairpin at the end of the hotspot 1 with residues such as Arg17 binding the ATP phosphate groups (figure 3*c*). This region thus provides a potential area for developing fragments from hotspot 1 further into the catalytic cleft. At hotspot 3, a possible hydrogen bond donor was observed matching the CAIR phosphate bind site, although the hydrophobic feature (yellow) of the hotspot is not well defined, unless when observed at contour 14, potentially explaining why no fragments were seen to bind in soaking experiments. The hotspot 4 at the edge of the catalytic site further beyond the ATP and CAIR binding sites and the additional hotspot 5 on the rear of the protein (figure 3*b*,*d*) could represent an allosteric site, the biological relevance of which requires further investigation.

### (d) Fragment screening using XChem and PanDDA with a diverse library

Fragment hits from the in-house fragment library all occupy the ATP indole pocket of MabPurC. To identify additional hotspots in PurC, we increased the chemical diversity of the library. Two libraries were employed. The first, the Leeds three-dimensional collection [25,26], comprises 125 fragments with fewer planar chemical groups and more natural-product-like scaffolds. Fragments from this library have high sp^3^ content providing more opportunities for elaboration. The second, the Diamond-SGC-iNext Poised Library, comprises 768 chemically diverse fragments with at least one functional group to allow a simplified chemical synthesis [27]. The hits from our in-house library (see above) were included as a positive control in the screening experiments.

The PanDDA method uses a collection of related crystallographic datasets to identify regions within individual sets that are statistical outliers, for example, indicating a changed conformational state due to ligand binding. A partial-difference or event map is created to reflect the density for the bound-state only. This is done by subtracting a proportion of the apo structure; the fragment-bound states are identified from the analysis of density distributions. The ensemble models are then refined with the help of standard resolution-dependent refinement procedures [16,17].

**Figure 4.**
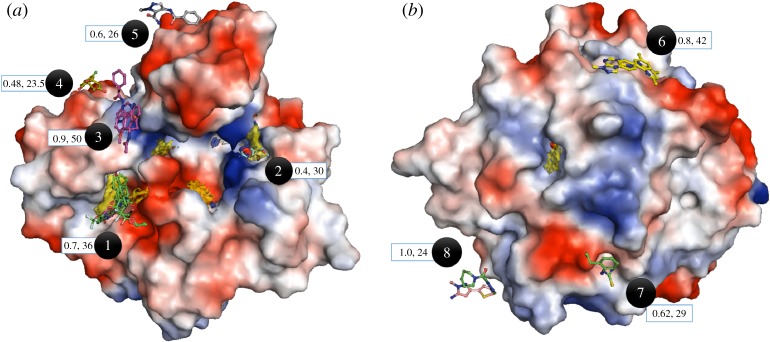
Fragment binding modes identified from screening of diverse fragment libraries by crystallography and analysis using PanDDA program. Hotspot maps are contoured at 14 and MabPurC protein is shown as surface electrostatic representation. (*a*) Front view of the protein with fragment binding sites 1–5 and representative hits. (*b*) Rear view of the protein having fragments binding sites 6–8 with representative hits. The average occupancies and B-factors corresponding to each site are also illustrated in the blue box.

Three hundred and four crystal structures were solved and 88 event maps identified by the PanDDA program were manually verified in *Coot* [28,29] and fragments were modelled and refined in 35 of them (figure 4). Almost 60% of all the identified hits occupy the first hotspot region of PurC corresponding to ATP adenylyl pocket. The positive controls (in-house library hits) were also analysed using the PanDDA method and found to adopt binding modes close to those previously determined in our laboratory.

In addition, several new hits and chemical scaffolds were identified at the same site from the more diverse fragments in the libraries used in this study. These compounds were bound with occupancies ranging from 1.0 to 0.7. This includes three-dimensional fragment **XC1,** in which the pyridine ring of the fragment makes hydrophobic and π-stacking interactions with the side chains of Leu19 and Arg17, respectively. Interestingly, the flexible chain with a halogen atom binds in the negatively charged sub-pocket, adjacent to the ATP adenine site at hotspot 1, where it interacts with side chains of Glu99 and Glu200 as well as active site water molecules (electronic supplementary material, figure S2A,B). This sub-pocket was not identified in previous fragment screening experiments.

Apart from numerous fragment hits hotspot 1 (ATP adenylyl site), the only other hotspot with an observed hit was number **XC2**, at the PurC active site cleft edge near the CAIR binding region. This fragment exhibits a low occupancy of 0.4 and forms H-bonds from its amino group to the side chain of Arg222 and from its sulfur atom to the amide of Gly108 and the hydroxyl group of Thr107 (see electronic supplementary material, figures S2C,D).

Interestingly, no fragment hits were observed at the predicted warm-spot region corresponding to the site 2—aspartic acid binding region and hotspot 3—substrate (CAIR) site of the PurC protein from either the in-house or PanDDA method indicating that these two sites may not be true hotspot regions. Indeed, the corresponding maps for the region are less evident when set at a higher cut-off (contour greater than 17), while hotspot maps corresponding to site 1 (ATP adenylyl pocket), 4 (edge of the active site cleft) and 5 (at the rear of the protein) remain unchanged even at a more stringent cut-off.

The remaining fragment hits identified from the PanDDA analysis do not involve any of the predicted hotspot regions but instead form weak interactions in shallow binding pockets, some near crystallographic symmetry axes. This is not surprising given that the PanDDA method is sensitive to low occupancy fragments resulting in weak density.

## 3. Discussion

The aim of screening the essential enzyme *M. abscessus* SAICAR synthetase (PurC) for fragment binding was to assess its usefulness as a target of antimicrobial drug discovery. We compare two different approaches to identify fragment binding sites. Screening the enzyme against an in-house fragment library of 960 fragments resulted in eight hits that were bound to the adenine pocket of the PurC ATP binding site. Interactions of these fragments were investigated by X-ray crystallographic and isothermal calorimetry (ITC) analyses. Fragment **2** was found to have a *K*_d_ of below 350 µM, indicating promising starting points for chemical elaboration. For PurC, we observe that all the fragments identified from standard difference maps occupy positions predicted by the computational hotspot mapping software [22].

Further fragment screening experiments of PurC were undertaken at the Diamond Light Source XChem facility using two chemically diverse fragment libraries. Resulting crystallographic datasets sets were density averaged and ensemble-modelled using PanDDA software. Again fragment binding site 1 predominated. Of the three interesting hits, two occupy a negatively charged sub-pocket adjacent to the ATP adenine-binding site as well as site 1, providing starting points for chemical merging or linking with previously identified fragments. A third fragment binds at a small pocket at the edge of the catalytic cleft adjacent to substrate CAIR binding region. If this interaction can be replicated, the fragment may be amenable for further intervention to develop a non-ATP competitive inhibitor of PurC enzyme. Thus, these experiments using diverse chemical libraries with three-dimensional chemical scaffolds together with the PanDDA method were able to identify new sub-pockets on which to build a future FBDD campaign.

The most challenging aspect of the XChem and PanDDA approach is the identification of many fragment hits that bind to protein sites with low occupancies (figure 4). Are these sites truly hot spots unidentified by the default contour level 17 in Fragment Hotspot Maps program [22], or are they weak binding sites routinely seen in crystals due to high concentrations of ligand molecules? This could be investigated by establishing whether such fragments maintain their binding modes and interactions when elaborated into larger chemical entities. This is a challenge, requiring extensive chemistry, which we will follow up in the future with PurC and other targets. On the other hand, they may be ‘warm spots’ where fragments are not bound with a sufficient gain of entropy to stabilize them when elaborated. These fragment hits may nevertheless be useful if sufficiently close to hotspots where a fragment is being elaborated to allow the design of molecules that exploit further interactions, the price of the loss of translational and rotational entropy already having been paid by the original fragment.

## 4. Material and methods

### (a) Crystallization of *M. abscessus* PurC

Initial screening of PurC protein for determining appropriate crystallization conditions involved the use of following commercially available sparse matrix screens: Drops containing 18 mg ml^−1^ of the protein in storage buffer (50 mM Tris–HCl pH 7.5, 150 mM NaCl) and reservoir at two different drop ratios: 0.3 µl : 0.3 µl and 0.6 µl : 0.3 µl (of protein: reservoir, respectively) were set up using a Mosquito crystallization robot (TTP Labtech), in 96-well sitting drop (MRC-2) plates. The drops were equilibrated against 80 μl of the corresponding reservoir solution at 19°C. The best diffracting crystals (1.5 Å resolution) were obtained from JCSG well H9, 0.2 M LiSO_4_, 0.1 M Bis–Tris pH 5.5, 25% PEG 3350 and this crystal condition was used for further experiments.

### (b) Co-crystallization of *M. abscessus* PurC protein with ATP

2 mM–5 mM final concentration of compound of ATP in DMSO/water was added to 18 mg ml^−1^ of PurC protein, mixed and incubated for 2 h on ice. Crystals were grown in the following condition: 0.2 M lithium sulfate, 21–28% PEG3350, 0.1 M Bis–Tris pH 5.5–6.5. The crystallization drops were set up at a protein to reservoir drop ratio of 0.3 µl : 0.3 µl, in a 96-well (MRC2) sitting drop plate, using a Mosquito crystallization robot (TTP Labtech) and the drops were equilibrated against 70 μl of the reservoir at 19°C.

### (c) Soaking of *M. abscessus* PurC native crystals with in-house fragment library

Crystals for this experiment were grown at 19°C in 48-well sitting-drop plates (Swiss CDI) in the following grid condition: 0.2 M Lithium sulfate, 21–28% PEG3350, 0.1 M Bis–Tris pH 5.5–6.5. Further, the crystals were picked and allowed to soak in a 4 μl drop containing reservoir solution and 10 mM fragments which were then equilibrated against 800 μl of the corresponding reservoir solution overnight at 19°C in a 24-well hanging drop vapour diffusion set-up.

### (d) X-ray data collection and processing

The PurC apo- and ligand-bound crystals were flash-cooled in cryo-protectant containing precipitant solution and 25% Ethylene glycol. X-ray datasets were collected by the rotation method and pixel array detectors at Diamond Light Source in the UK, using beamlines I03, I04, I24 and I04-1 at a wavelength of 0.979 Å (0.93 Å at I04-1), and at the Soleil French National Synchrotron facility at wavelength 0.979 Å. Datasets comprised a total oscillation of 210°–240° and oscillation angles of 0.15–1° per image, and total dataset exposure of 105–192 s at Diamond, and 105–120 s at Soleil. The diffraction images were processed using AutoPROC [30], using XDS [31] for indexing, integration, followed by POINTLESS [32], AIMLESS [33] and TRUNCATE [34] programs from CCP4 Suite [35] for data reduction, scaling and calculation of structure factor amplitudes and intensity statistics. All PurC crystals belonged to space group P2_1_ and consisted of one protomer in the asymmetric unit.

### (e) Structure solution and refinement

The *M. abscessus* PurC apo structure was solved by molecular replacement using PHASER [36] with the atomic coordinates of *M. abscessus* PurC at 1.85 Å (PDB entry: 3R9R, Seattle Structural Genomics Consortium for Infectious Diseases) as search model and PurC ligand-bound structures were solved by molecular replacement with the atomic coordinates of the solved *M. abscessus* PurC apo structure as search model. Structure refinement was carried out using REFMAC [37] and PHENIX [38]. The models obtained were manually re-built using COOT interactive graphics program [28] and electron density maps were calculated with 2|*F*_o_| − |*F*_c_| and |*F*_o_| − |*F*_c_| coefficients. Positions of ligands in the protein active site and water molecules were located in difference electron density maps and OMIT difference maps |mFo − DFc| [39] were calculated and analysed to further verify positions of fragments and ligands.

### (f) Extended fragment library crystallographic screening using XChem and PanDDA

The crystals used in this study were grown at 19°C in 348-well 3 drop, sitting drop plates (Swiss CI) in the following grid condition: 0.2 M Lithium sulfate, 21–28% PEG 3350, 0.1 M Bis–Tris pH 5.5–6.5 using PurC protein at a concentration of 18 mg ml^−1^ equilibrated against 40 µl reservoir. Apo crystals were allowed to soak in 30–50 mM fragments for 1 h. Crystal soaking, harvesting, mounting and data collections were performed at the Diamond Light Source I04-1 beamline through the XChem facility workflow [14]. After molecular replacement and refinement of the initial model, the resulting maps were analysed by PanDDa [16] followed by model building using Coot [28]. The ensemble models were then refined with the help of standard resolution-dependent refinement procedures [16,17].

## Supplementary Material

Supplementary Data

## Data accessibility

This article has no additional data.
