# Supplementary material for: Structure-guided fragment-based drug discovery at the synchrotron: screening binding sites and correlations with hotspot mapping
Source: Philos Trans A Math Phys Eng Sci. 2019 Apr 29;377(2147):20180422. doi: 10.1098/rsta.2018.0422 (PMC6501894; doi:10.1098/rsta.2018.0422)
Supplement: Supplementary Data [file rsta20180422supp1.docx]

**Supplementary data**

**Structure-guided Fragment-based Drug Discovery at the Synchrotron: New Developments in Screening Binding Sites and Correlations with Hotspot Mapping**

Sherine E. Thomas^1^, Patrick Collins^2^, Rory Hennell James^1,3^, Vitor Mendes^1^, Sitthivut Charoensutthivarakul^4^, Chris Radoux^5^, Chris Abell^4^, Anthony G. Coyne^4^, Andres R. Floto^6,7^, Frank von Delft^2,3,9^ and Tom L. Blundell^1^

Philosophical Transactions of the Royal Society

Article DOI 10.1098/rsta-2018-0422

We use the enzyme PurC, involved in the biosynthesis of purine nucleotides (1), as an example of a protein target for fragment screening. Here we provide the background experimental data for the enzyme, its validation as a target, comparison with the human orthologue, its cloning, expression and characterisation, two biophysical approaches used to select fragments that bind PurC, and a figure giving details of two hits. These provide the context for the comparative study of a traditional X-ray difference Fourier approach and the new XChem approach at the Diamond synchrotron and the PanDDA method for analysing multiple data sets, described in in the main text.

**Background to PurC biochemistry**

PurC, or phosphoribosylaminoimidazole-succinocarboxamide (SAICAR synthetase), catalyzes the eighth step of *de novo* purine biosynthesis pathway in bacteria and fungi. PurC mediates the ligation of L-aspartate (Asp) with the substrate, 5-amino-1-(5-phospho-D-ribosyl) imidazole-4-carboxylate (CAIR) in the presence of ATP and Mg^2+^ to form SAICAR (Figure 1A). The enzyme was first described in 1959 (2), further characterised in 1962 (3), and the first structure of a PurC enzyme determined from *Saccharomyces cerevisiae* (4). Since then, PurC structures from 12 different species have been elucidated in apo- and ligand-bound forms.

Although most bacterial PurC enzymes exist as homo-dimers (5-7), PurC enzymes from *Mycobacterium abscessus* and *Saccharomyces cerevisiae* function as monomers (4). PAICS, the human orthologue of PurC, exists in a bi-functional octameric form, combining both 5-aminoimidazoleribonucleotide carboxylase (AIR carboxylase) and phosphoribosylaminoimidazole-succinocarboxamide synthetase (SAICAR synthetase) activities (8).

Although purine biosynthesis is an extremely important biosynthetic pathway necessary for production of ATP and GTP for energy metabolism and the synthesis of DNA and RNA, it is an under-exploited antimicrobial target. Studies with *Escherichia coli*, *Bacillus cereus* and *Acinetobacter baumannii* have shown that bacterial growth in the gut, blood and lungs respectively requires purine biosynthesis (9-11). The differences in function and subunit organization of bacterial and human SAICAR synthetases, together with essentiality in bacteria, suggest the potential for development of novel anti-microbial therapies by selective inhibition of bacterial PurC.

**Comparison of *M abscessus* PurC with the *Homo sapiens* ortholog *Hs*PAICS**

To confirm that *Mycobacterium abscessus* PurC (*Mab* PurC) is sufficiently different from the *Homo sapiens* ortholog *Hs*PAICS and that cross-reactivity of a drug was unlikely, the apo structures were compared. *Hs*PAICS (23.5 % sequence identity with *Mab* PurC) forms an octamer, with a central ring of C-terminal AIR carboxylase (AIRc) domains and an outer ring of N-terminal SAICAR synthatase (PurC) domains (**Figure S1 A**).

The PurC domain of one *Hs*PAICS protomer and *Mab* PurC were superimposed for comparison (**Figure S1 B**). *Mab* PurC is approximately 30 amino acids longer than the SAICAR domain of *Hs*PAICS with small insertions at the rear of the β-sheet above the ATP site (*) and the bottom of the α-helix in the CAIR site (†). The C-terminus of *Mab* PurC folds back on the rear of the protein rather than extending towards the AIR carboxylase domain position as in *Hs*PAICS. There is also a larger insertion at the N-terminus of the most C-terminal helix, which extends the helix and adds a poorly-resolved flexible loop and an additional helix (‡). These insertions are not in regions thought to be catalytically important but they may have regulatory implications (12).

Several *Mab* PurC residues that interact with ATP at Hotspot 1 differ in *Hs*PAICS (**Figure S1 C**). The most surprising differences are R17, which makes charge-charge interactions with the phosphates of ATP but is replaced by a threonine in *Hs*PAICS, and H69, which makes a hydrogen bond with the amine of the adenyl group but is replaced by alanine in *Hs*PAICS. The other differing residues make interactions via backbone amines and carbonyls so the changes probably have little effect on this interaction. Compounds that interact with these non-conserved residues should be able to discriminate between the pathogen *Mab* PurC and the patient *Hs*PAICS. The residues at Hotspot 4, however, are more conserved across the *Hs*PAICS and *Mab* PurC. The corresponding interactions of fragment hits mediated by R222 and T107 at this allosteric site are therefore conserved between the two orthologs while residue Gln235 of Mab PurC has been substituted by M244 in HsPAICS. Further, the region ^235^QDSF^238^ corresponding to the loop, spanning the edge of the catalytic site of Mab PurC, has been substituted with ^244^MVKK^247^ in HsPAICS (**Figure S1 D**). The fragment could be elaborated to make use of the interactions in this region to develop selective non-ATP competitive inhibitor of Mab PurC.


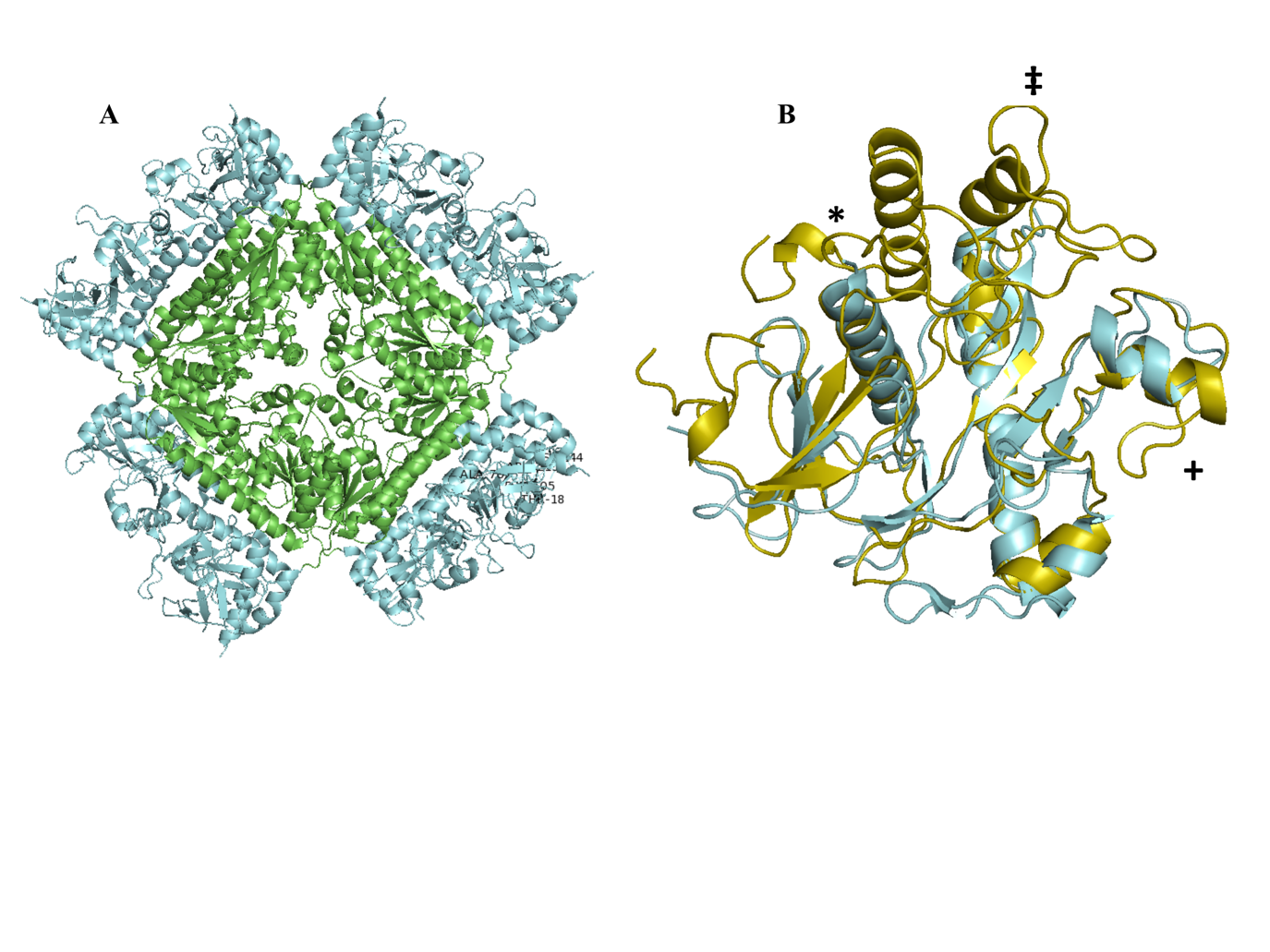


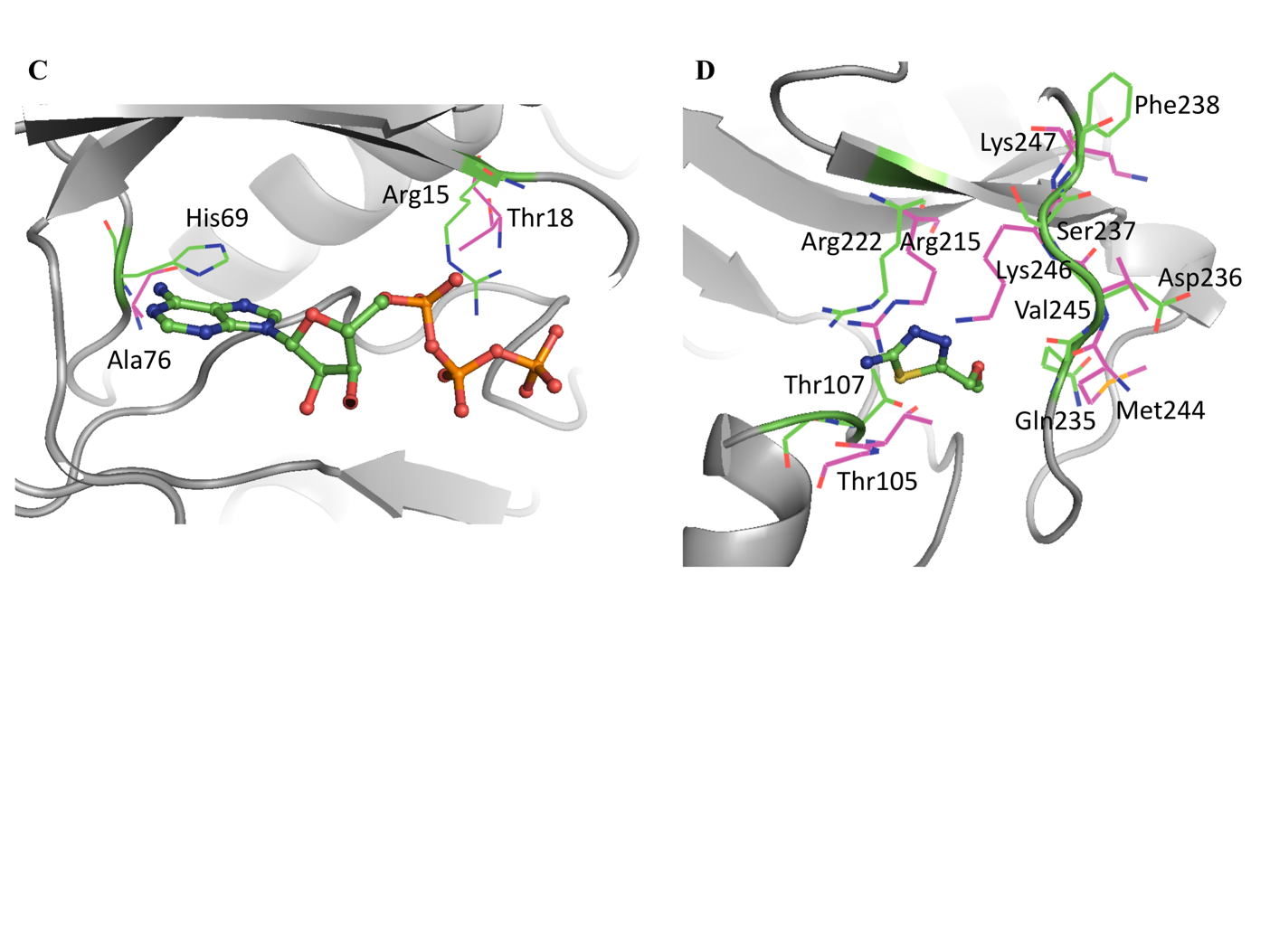


**Figure S1:** **A)** *HsPAICS octamer (PDB* ***2H31****) showing the inner AIR carboxylase domain (green) and the outer SAICAR synthetase (PurC) domain in blue* ***B)*** *Superposition of one protomer of HsPAICS SAICAR synthetase domain (blue) and Mab PurC (gold). Insertions (*, † & ‡) in Mab PurC relative to HsPAICS are indicated.* ***C)*** *Hotspot 1 (ATP site of PurC). Superposition of the Mab PurC (green) and HsPAICS (magenta) ATP sites, showing non-conserved residues in line representation. The most surprising substitutions (R15 to T18 and H69 to A76) are indicated.* ***D)*** *Fragment hit (green stick) at hotspot 4 (an allosteric site of PurC-identified in this study) and the conserved residues that interacts with the fragment, R222 and T107, which are conserved between Mab PurC (green line) and HsPAICS (magenta line). However, residue Gln235 of Mab PurC has been substituted by M244 in HsPAICS as well as region ^235^QDSF^238^ of the loop has been substituted with ^244^MVKK^247^ in HsPAICS. The fragment could be elaborated to make use of interactions in this loop region to develop selective non-ATP competitive inhibitor of Mab PurC.*

**Amplification of *purC* gene from *Mycobacterium abscessus* genomic DNA**

Genomic DNA sample was obtained from *Mycobacterium abscessus* (ATCC 19977). The stock of genomic DNA (6ng/μl) was diluted using sterile water to make a working concentration of 0.6ng/μl. The PurC gene (MAB_0689) was amplified using the following primers (Sigma):

Forward Primer: 5’-ATTCCATGGTGCGTCCTTCGCTGTCCGATTAC-3’

Reverse Primer: 5’-TATCTCGAGTCACGCCGACGGGCCAATCC-3’

The following thermocycle program was used: stage 1 X 1cycle: activation of polymerase at 95^o^C for 2min, stage 2 X 35 cycles: denaturation at 95^o^C for 20 sec, annealing at 70^o^C for 20 sec, extension at 70^o^C for 45 sec. This was followed by stage 3: final extension at 70^o^C for 10 min.

**Molecular Cloning**

The purified PCR product and a PHAT2 *E. coli* expression vector containing Ampr and non-cleavable N-terminal His-tag were then subjected to restriction digestion with *Nco1* and *XhoI* restriction endonucleases (ThermoScientific). The ligation of digested insert and vector was performed using T4 DNA ligase (New England Biolabs), by incubation at room temperature for 10 minutes. The ligation product was transformed in to *E. coli* DH5α competent cells by heat-shock method and plated on LB agar-kanamycin plates and incubated at 37°C. Single colonies were randomly picked on the following day and inoculated in LB media with kanamycin (30 μg/mL) and grown overnight at 37°C. Plasmids from the resulting cultures were isolated and purified (ThermoScientific GeneJet Plasmid Miniprep Kit). The integrity of the clones was confirmed by sequencing (DNA Sequencing Facility, Department of Biochemistry, University of Cambridge, UK).

**Expression and Purification of N-His-PurC full length**

*E. coli* BL21 (DE3) strains containing N-His-PurC PHAT2 plasmids were grown overnight at 37°C in LB-media containing Ampicillin (100 μg/ml). This seed-stage culture was used to inoculate 6 shake flasks containing 1 L each of 2XYT media with Ampicillin (100 μg/mL) until optical density (A_600nm_) reached 0.6. Expression of recombinant construct was induced by the addition of Isopropyl β-D-1-thiogalactopyranoside (IPTG) to a final concentration of 0.5 mM and further allowed to grow at 18 °C for 16 hrs. Cells were harvested by centrifugation at 4°C for 20 min at 4200 rpm and the pellet was re-suspended in buffer A (50mM Tris-HCl pH 7.5, 350 mM NaCl, 20 mM Imidazole). 10μg/mL DNaseI, 5 mM MgCl_2_ and 3 protease inhibitor cocktail tablets (New England Biolabs) were added to the cell suspension. The cells were lysed by sonication (Branson) and the lysate was clarified by centrifugation at 4°C for 40 min at 25,568g. The clarified lysate was filtered using a 0.45 μm syringe filter and passed through a pre-equilibrated (with buffer A), 10mL pre-packed Nickel- sepharose column (HiTrap IMAC FF, GE Healthcare). The column was washed with 5 column volumes of buffer A and the bound protein was eluted as 2 x 12mL elutes using buffer B (50 mM Tris-HCl pH 7.5, 350 mM NaCl and 500 mM Imidazole). The protein was analyzed on a 15% SDS-PAGE gel.

Elutes 1&2 from HiTrap IMAC column were pooled and subjected to dialysis against 2 L of buffer C (50 mM Tris-HCl pH 7.5, 350 mM NaCl) overnight at 4°C. After overnight dialysis the protein was concentrated to 3 mL using a 10 kDa centrifugal concentrator (Sartorius Stedim) and loaded onto a pre-equilibrated (with buffer D: 50 mM Tris-HCl pH 7.5, 150 mM NaCl) 120 mL Superdex200 16/600 column (GE Healthcare). 2mL fractions were collected and analyzed on a 15% SDS-PAGE gel. Fractions corresponding to pure PurC protein were pooled and concentrated to 26 mg/mL, flash frozen in liquid nitrogen ad stored at -80°C.

**Screening of in-house fragment library by Thermal Shift Assay**

Thermal Shift Assays were performed to determine binding of *M. abscessus* PurC with fragments from an in-house fragment library. The assays were carried out in a 96-well format with each well containing 25 μL of reaction mixture of 20 μM PurC protein in buffer (100 mM Hepes pH 7.5, 150 mM NaCl, 1 mM MgCl_2_), 5 mM compound, 5% DMSO and 5X Sypro orange dye. Appropriate positive (Protein, DMSO and ATP) and negative (Protein, DMSO only) controls were also included. The measurements were performed in a Biorad-CFX connect thermal cycler using the following program: 25°C for 10mins followed by a linear increment of 0.5°C every 30 sec to reach a final temperature of 95°C. The results were analyzed using Microsoft excel.

**Isothermal Titration Calorimetry**

ITC experiments were carried out in buffer containing 100mM HEPES pH 7.5, 150 mM NaCl and 1 mM MgCl_2_. Protein was subjected to overnight dialysis against this buffer prior to the experiments and subsequent dilutions of protein and ligands used the remaining buffer. Generally, ligands were titrated at a concentration of 5-10 mM fragment hits from the in-house library against 100 μM PurC protein in a Malvern MicroCal iTC200 instrument. To correct for enthalpy of dilution ligands were also titrated against buffer and the peak values of this run subtracted from the experimental run as a reference. When ligands were dissolved in DMSO an appropriate concentration of DMSO was added to other solutions. Data were analysed using the Origin software (OriginLab, Northampton, MA, USA).

***Table 1:*** *Hits identified from screening of the in-house fragment library against Mab PurC. The corresponding ∆T_m_ (^o^C) values from DSF and K_d_ (µM) from ITC measurements are also shown.*

| **Fragment** | **Structure** | **M.W. (Da)** | **∆T_m_ (^o^C)** | **K_d_ (µM)** |
| --- | --- | --- | --- | --- |
| **1** |  | 128.155 | + 1.38 | 709 |
| **2** |  | 146.15 | + 1.63 | 347 |
| **3** |  | 129.6 | + 1.0 | 971 |
| **4** |  | 135.2 | + 2.25 | 315 |
| **5** |  | 169.6 | + 1.63 | ND |
| **6** |  | 135.1 | + 2.13 | 794 |
| **7** |  | 180.2 | + 2.25 | 178 |
| **8** |  | 164.14 | + 1.88 | ND |

**Examples of fragment hits identified from XChem and PanDDA method**


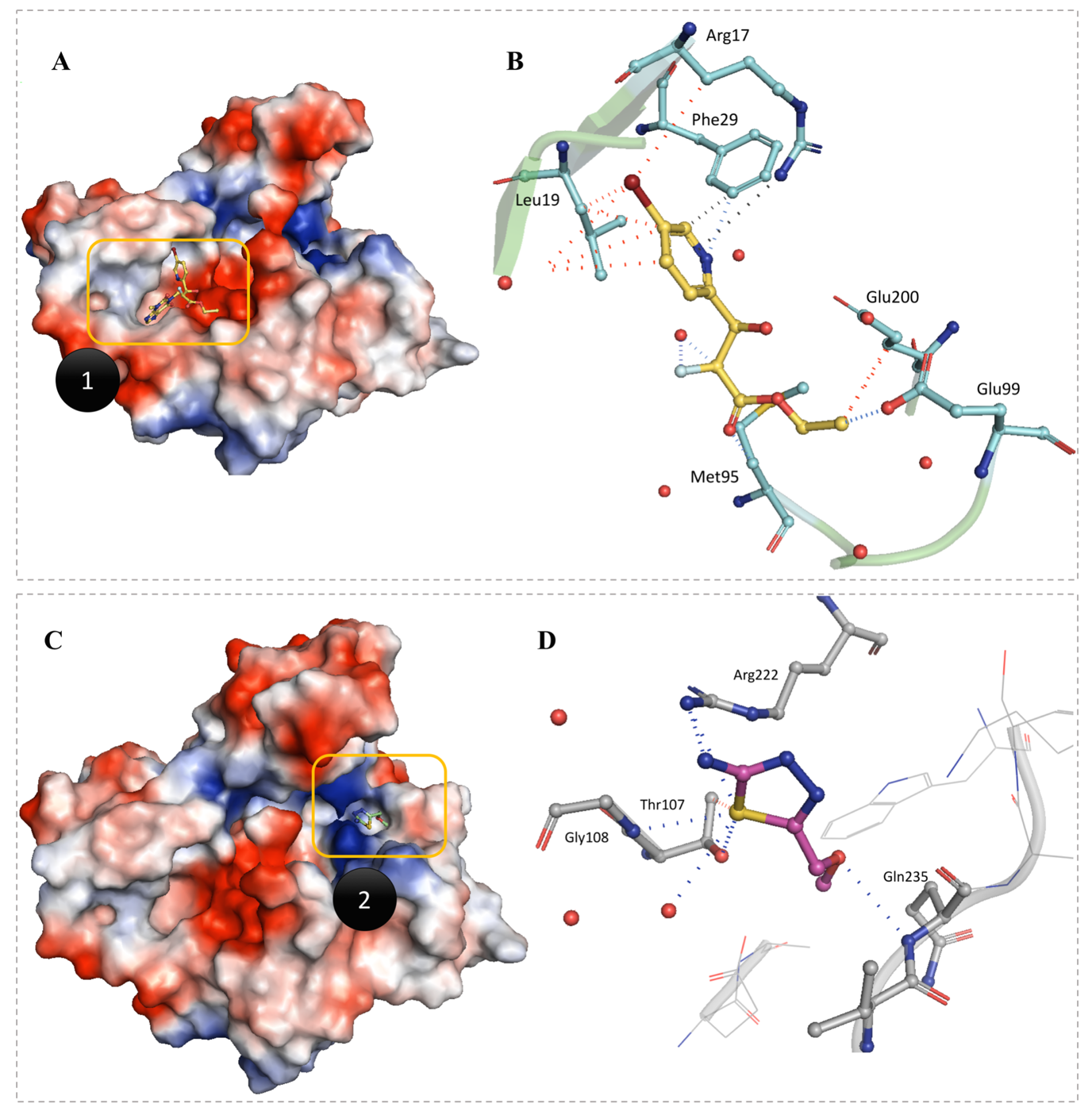


***Figure S2****: Examples of fragment hits identified from XChem and PanDDA method.* ***A)*** *Crystal structure of MabPurC, shown as surface electrostatic model, in complex with 3D* ***fragment XC1*** *(yellow stick) binding at a negatively charged sub-pocket adjacent to the ATP adenine ring* ***B)*** *Interactions of* ***fragment 3*** *(shown as yellow stick) with PurC amino acids residues (blue) and water molecules (red).* ***C)*** *Crystal structure of MabPurC, shown as surface electrostatic model, in complex with* ***fragment XC2*** *(green stick), which binds at the edge of the PurC active site cleft adjacent to the CAIR binding region and* ***D)*** *Interactions of the fragment (pink stick) with amino acid residues (grey) and water molecules (red). Hydrogen bonding interactions are depicted in blue, π- interactions in black and hydrophobic contacts in red dotted lines respectively.*

**References Supplementary**

1. Ashihara BAMaH. Purine and Pyrimidine Nucleotide Synthesis and Metabolism: The American Society of Plant Biologists; 2002.

2. Lukens LN, Buchanan, J.M.,. Biosynthesis of the purines. XXIII. The enzymatic synthesis of N-(5-amino-1-ribosyl-4-imidazolylcarbonyl)-L-aspartic acid 50 - phosphate. . J Biol Chem 1959;34:1799–805.

3. Miller RW, Buchanan, J.M. Biosynthesis of the Purines: XXVII. N-(5-amino-1-ribosyl-4-imidazolylcarbonyl)-L-aspartic acid 5'-phosphate Kinosynthetase. The Journal of biological chemistry. 1962;237:485–90.

4. Levdikov VM, Barynin VV, Grebenko AI, Melik-Adamyan WR, Lamzin VS, Wilson KS. The structure of SAICAR synthase: an enzyme in the de novo pathway of purine nucleotide biosynthesis. Structure. 1998;6(3):363-76.

5. Ginder ND, Binkowski DJ, Fromm HJ, Honzatko RB. Nucleotide complexes of Escherichia coli phosphoribosylaminoimidazole succinocarboxamide synthetase. The Journal of biological chemistry. 2006;281(30):20680-8.

6. Zhang R, Skarina T, Evdokimova E, Edwards A, Savchenko A, Laskowski R, et al. Structure of SAICAR synthase from Thermotoga maritima at 2.2 angstroms reveals an unusual covalent dimer. Acta crystallographica Section F, Structural biology and crystallization communications. 2006;62(Pt 4):335-9.

7. Wolf NM, Abad-Zapatero C, Johnson ME, Fung LW. Structures of SAICAR synthetase (PurC) from Streptococcus pneumoniae with ADP, Mg2+, AIR and Asp. Acta crystallographica Section D, Biological crystallography. 2014;70(Pt 3):841-50.

8. Li SX, Tong YP, Xie XC, Wang QH, Zhou HN, Han Y, et al. Octameric structure of the human bifunctional enzyme PAICS in purine biosynthesis. Journal of molecular biology. 2007;366(5):1603-14.

9. Samant S, Lee H, Ghassemi M, Chen J, Cook JL, Mankin AS, et al. Nucleotide biosynthesis is critical for growth of bacteria in human blood. PLoS Pathog. 2008;4(2):e37.

10. Vogel-Scheel J, Alpert C, Engst W, Loh G, Blaut M. Requirement of purine and pyrimidine synthesis for colonization of the mouse intestine by Escherichia coli. Appl Environ Microbiol. 2010;76(15):5181-7.

11. Wang N, Ozer EA, Mandel MJ, Hauser AR. Genome-wide identification of Acinetobacter baumannii genes necessary for persistence in the lung. MBio. 2014;5(3):e01163-14.

12. Manjunath K, Jeyakanthan J, Sekar K. Catalytic pathway, substrate binding and stability in SAICAR synthetase: A structure and molecular dynamics study. J Struct Biol. 2015;191(1):22-31.
